# Research and Application of High-Pressure Rotary Jet Method in the Seepage Treatment of Heavy Metal Tailing Ponds of Southwest China

**DOI:** 10.3390/ma16093450

**Published:** 2023-04-28

**Authors:** Mengjia Liang, Chunzheng Jin, Jiwu Hou, Mengyuan Wang, Yanping Shi, Zichao Dong, Xianyu Yang, Jianwei Zhou, Jihua Cai

**Affiliations:** 1 Faculty of Engineering, China University of Geosciences, No. 388, Lu Mo Road, Wuhan 430074, China; suju_elfbylmj@cug.edu.cn (M.L.);; 2 School of Environmental Studies, China University of Geosciences, No. 388, Lu Mo Road, Wuhan 430074, China

**Keywords:** heavy metal, tailing, karst, seepage treatment, high-pressure rotary jet, permeability coefficient

## Abstract

The developed karst caves may become the seepage channels of heavy metal to the soil and underground water in Southwest China. Therefore, it is necessary to apply effective seepage treatments to the base of heavy metal tailing reservoirs. This paper addressed the high-pressure rotary jet technology and slurry systems used in the seepage treatment of the deep tailing sand of the Shenxiandong tailing pond located in Southwest China. In this study, the factors of fluidity, initial and final setting times, compressive strength, and permeability coefficient of the slurry were conducted. The mechanism analysis was investigated by X-ray diffraction (XRD), scanning electron microscope (SEM), and inductively coupled plasma-mass spectrometry (ICP-MS). Three different types of slurry systems were proposed, and the permeability coefficients of the solidification body following 28 days of curing were less than 1 × 10^−7^ cm/s. The concentrations of Pb and Zn in the slurry system containing bentonite were reduced by 26.2% and 45.7%, respectively. In the presence of slaked lime and fly ash, the concentrations of Pb and Zn could be reduced by 26.8% and 30%, respectively. A total of 2142 high-pressure rotary jet piles were completed by the high-pressure rotary jet method in the field trial. The diffusion radius of these piles was over 1 m. Following 28 days of curing, the solidification body’s compressive strength was 7.45 MPa and the permeability coefficient was 6.27 × 10^−8^ cm/s. Both the laboratory and on-site trials showed that this method produced a good pollution barrier effect, which could prevent the diffusion of heavy metal into the adjacent underground water through the karst caves. It is also an effective way of engineering technology concerning heavy metal pollution control that occurs in tailing ponds.

## 1. Introduction

Over 150 types of minerals have been discovered in Southwest China, but some non-ferrous metal mineral resources are abundant now. Yunnan Province is known as China’s “non-ferrous-metal kingdom” because of its rich non-ferrous-metal resources, of which tin, lead, and zinc reserves rank among the top three in China [1]. Guizhou Province is one of the world’s important mercury (Hg)-production centers. To date, at least 12 large and super-large Hg mines (e.g., Wanshan, Wuchuan, Lanmuchang, and Danzhai) have been discovered in Guizhou Province [2]. According to the 12th Five-Year Plan for the Comprehensive Prevention and Control of Heavy Metal Pollution issued by the Ministry of Environmental Protection of the People’s Republic of China, there are 31 key prevention and control areas in Southwest China of the 138 nationals, including 11 in Yunnan. The tailing ponds in these metal mines pose a great potential threat to the surrounding fragile ecological environment due to the karst landscapes in Southwest China. In 2011, after tailing slag pollution occurred in a tailing pond in Songpan County, Sichuan Province, it caused groundwater pollution in Mianyang City, which was located 300 km away from the accident site [3]. Therefore, concerning the potential threat posed by the tailing ponds located in the metal mines in Southwest China, the studies of pollution source control, remediation technology, and equipment used for common minerals in these tailing ponds have become urgent. With the gradual increase in the nation’s awareness of environmental protection factors, China Nonferrous Metals Industry Association (CNIA) promulgated the “Non-Ferrous-Metal Industry Solid Waste Pollution Control Standard” (SB 5085-85) in 1985. It helped to supervise and prevent the pollution sources of tailing ponds pollute surrounding soil and groundwater sources by seepage. After the release of numerous environmental protection regulations and standards, the tailing-pond seepage control engineering technology has entered a benign development stage in China. The environmental protection standard of tailing ponds had shifted from the primary stage of “permeability coefficient *K* is not greater than 1.0 × 10^−5^ cm/s” to “*K* is not greater than 1.0 × 10^−7^ cm/s”. The tailing leakages, acid mine drainage, and other environmental problems have posed a threat to human life. Therefore, further scientific, effective, and economical prevention and control measures need to be proposed. These measures also can be coupled with rigorous technical testing to ensure that tailing ponds cannot be leaked.

High-pressure rotary jet grouting is a useful and effective method that is widely used in the field of geotechnical engineering [4]. The piles by this method can reduce settlement outcomes and increase the bearing capacity of existing and new foundations, support open and underground excavations, and create water cut-offs for dams [5].

The main principle of this method is to attach a nozzle to the drill pipe, and the cement slurry is sprayed with high pressure to the soil. The soil can be cut and mixed with cement slurry by rotating and lifting the drill pipe, and finally, a solid and impermeable consolidation body will be formed through physical and chemical means [6]. According to the number of injected fluids, the three types of jet-grouting methods and their application fields are shown in Table 1.

The construction technology, equipment, and grouting materials of high-pressure rotary jet technology are also rapidly developing according to the requirements of engineering, environmental quality, and protection. Some high-pressure rotary jet grouting drilling rigs are equipped with monitoring system modules, a pressure control system, and a spoil discharge system. During the jet grouting process, the construction parameters, such as water pressure, grouting rate, and retracting velocity, can be automatically detected, recorded, and controlled [4,11]. Cement-based grouting materials are widely used in complex and diverse engineering fields for their advantages of a low price, non-toxic, high-flow dynamic, and high-strength capacity [16,17,18]. Setting time is a key consideration regarding cement-based grouting materials, which seriously affects the overall grouting effect [19,20]. Therefore, in relation to all these problems, the performance of slurry with all aspect requirements needs further research.

**Figure 1:**
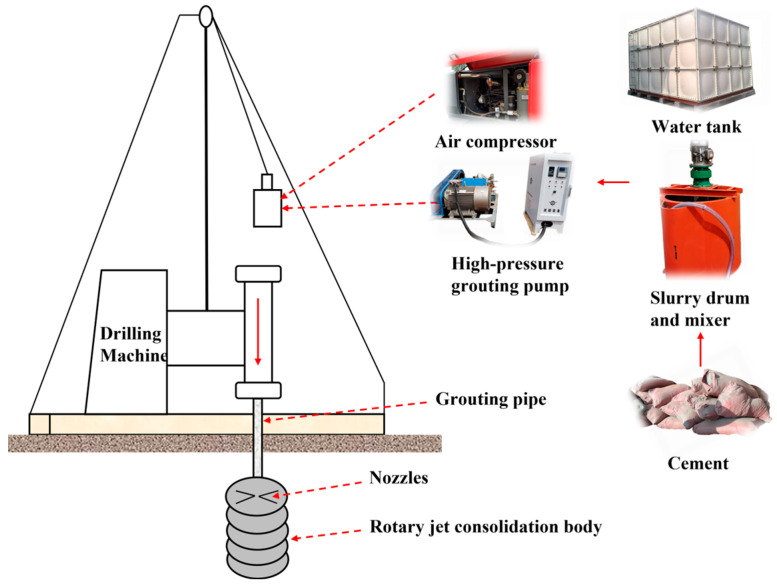
The double-pipe method high-pressure rotary jet grouting diagram.

As previously mentioned, the Shenxiandong tailing pond was closed in 2015, and no protective measures were taken to secure the top section of the tailing pond in Jianshui County, Yunnan Province, Southwest China. According to a site survey, the sealed field barrier area was approximately 9000 m^2^. For some heavy metal tailing leakage areas affecting the surrounding soil and groundwater, including other environmental problems, it is an urgent requirement to adopt good anti-seepage and plugging measures. The simple construction process, brief construction period, small investment, great practicability, and it does not cause environmental pollution are the advantages of the double-pipe method (Figure 1). So, it was used in the Shenxiandong tailing pond to solve the leakage problem of a tailing pond at present.

The high-pressure rotary jet grouting slurry is mixed with foundation soil and tailing sand to form the high-pressure rotary jet composite pile. It not only can take advantage of the strength of the natural foundation soil, but also have the function of strengthening the foundation, waterproof, and anti-seepage qualities. The high-pressure rotary jet slurry can also passivate the heavy metal elements present in the tailing, effectively decrease, or eliminate the concentration of pollutants present, and reduce the risk of pollutants to human health and the external environment.

Based on the environmental geological survey conducted on the Shenxiandong lead–zinc tailing pond in Jianshui County, Yunnan Province, the general state of the tailing pond project and surrounding soil pollution and analysis of the basic performance of tailing were investigated. This paper assessed the high-pressure rotary jet equipment, technology, and slurry systems used in the seepage treatment of the heavy metal tailing reservoir. To create slurry systems suitable for high-pressure rotary injecting technology in the deep tailing sand in the Shenxiandong tailing pond located in Southwest China, experimental tests on the fluidity, initial and final setting times, compressive strength, and permeability coefficient, together with XRD, SEM, and ICP-MS analyses, were conducted. Three types of slurry systems were proposed: a simple P.O 42.5 cement slurry system, a simple specially developed cementitious material slurry system, and a complex P.O 42.5 cement slurry system containing a water-reducing agent and solidification/stabilization agents. The high-pressure rotary jet technology for the seepage treatment of heavy metal tailing ponds was introduced. The field trial of the high-pressure rotary jet method was conducted at the Shenxiandong tailing pond, in the Jianshui County of Yunan Province, in Southwest China.

## 2. Materials and Methods

### 2.1. Experimental Materials

#### 2.1.1. Tailing

**Figure 2:**
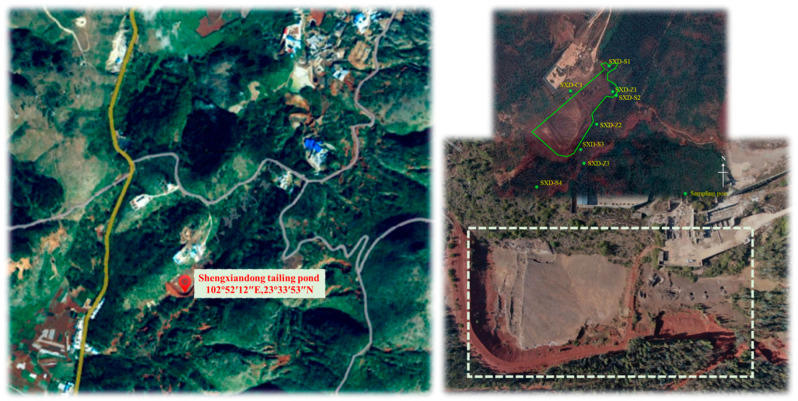
The geographical location of Shenxiandong tailing pond and distribution of sampling points.

The lead–zinc tailing sand used in the experiment was collected from the Shenxiandong tailing pond in Jianshui County, Yunnan Province (102°52′12″ E, 23°33′53″ N). A total of 15 soil samples located close to the approximately 18,000 m^2^ tailing pond were collected by the Beijing Mining and Metallurgy Technology Group Co., LTD. The sampling locations are depicted in Figure 2. Among them, SXD-S1~S4 were the surface sample points (4 samples in total), SXD-Z1~Z3 were 3 column samples (8 soil samples in total), SXD-C1 was a soil sample point on the concentrator side, SXD-B1~B2 were two background points, and the sampling horizon was 0–1.0 m.

#### 2.1.2. Additives

Sodium aluminate (NaAlO_2_) was selected as an effective accelerator, which was used to facilitate concrete setting and strength gain speed [21]. Lignin water-reducing agent was used to obtain high fluidity concrete with a low water–cement ratio, and favorable characteristics such as good water retention and slump retention [22,23]. Bentonite, with montmorillonite as the main mineral component, as a representative nonmetallic clay material, has been proven to be efficient in the removal of heavy metal ions. Moreover, bentonite has some outstanding physicochemical properties including large specific surface area, chemical stability, low cost, high porosity, and ubiquitous availability [24,25,26,27]. The slaked lime has a good solidifying effect on the heavy metal and sulfates in the tailing [28]. Fly ash has a homogenization effect and lubrication effect brought by its shape, the hydration products of its can also fill the gaps in the material, thereby increasing the strength and improving the impermeability [29,30].

In the basic experiment, orthogonal experiments were conducted on NaAlO_2_, lignin water-reducing agent, hydrated lime, and fly ash. The optimal ratio with a flow degree greater than 150 mm (to meet pumpability) and final setting time within 400 min was selected by the orthogonal experiment, as detailed in Table 2 and Table 3. The addition of the additives was calculated relative to the quality of the cement.

Ordinary Portland cement P.O 42.5 was bought from Jiuqi Building Materials Co., Ltd., Weifang, China; the curing agent was specially developed; bentonite was bought from Jiezuan Co., Ltd., Nanjing, China; NaAlO_2_ and lignin water-reducing agent were bought from Sinopath Chemical Reagent Co., Ltd., Shanghai, China; hydrated lime was bought from Shanghai Jianghu Titanium White Chemical Products Co., Shanghai, China; and fly ash was bought from Hejin Longjiang Fly Ash Development and Utilization Co., Ltd, Hejin, China.

### 2.2. Experimental Methods

#### 2.2.1. X-ray Diffraction (XRD) Test of Lead–Zinc Tailing

Three randomly selected lead–zinc tailing samples were taken at Shenxiandong. The tailing samples passed through a 200-mesh screen were dried in an electric blast dryer at 180° for 3 h, which were analyzed for composition using an X-ray diffractometer (X’Pert PRO DY2198, Panaco, The Netherlands).

#### 2.2.2. Experiments on the Initial and Final Setting Times of Cement Slurry

To optimize the formulation of the accelerator, a setting time experiment for the cement slurry was conducted. The initial and final setting times of the cement slurry were tested by Vicat apparatus, in accordance with the national standard of “General Silicate Cement” (GB 175-2007). The tested cement paste was loaded into the mold to the Vicat apparatus initial setting test needle sinking to (4 ± 1) mm from the bottom plate for the time required for the initial setting. After the initial setting, the test mold together with the slurry was taken down from the glass plate in a pan way and turned over 180° on the glass plate, when the ring attachment on the Vicat apparatus final setting test needle does not leave traces on the test body, the final setting state is reached, and this time is the final setting time.

#### 2.2.3. Fluidity Test of Cement Slurry

To test whether the cement slurry meets the pumpability standard, a fluidity experiment was designed. Conical dies with an upper opening diameter of 36 mm, a lower opening diameter of 60 mm, a height of 60 mm, and dimensions of 400 mm × 400 mm × 5 mm were used to measure the fluidity. The fluidity test was carried out following the Chinese National Standard “Test method for fluidity of cement mortar” (GB/T 2419-2005). First, the cement slurry was poured into a conical round mold that was placed on a horizontal glass plate. Then, the conical round mold was lifted vertically, and the maximum diameter in two mutually perpendicular directions was measured on the glass plate. Finally, the average value of the two directions was taken. Each experiment was repeated twice, and the average value was taken to express the fluidity of cement slurry.

#### 2.2.4. Core Permeability Coefficient Test

**Figure 3:**
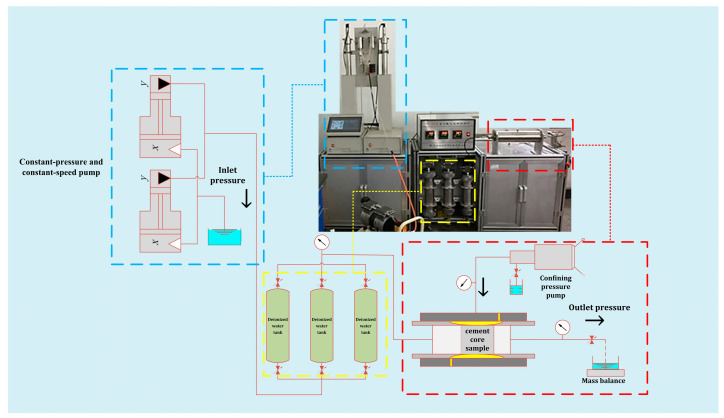
Core-flooding apparatus.

Due to the impermeability requirements of the tailing ponds, the standard permeability coefficient *K* should be less than or equal to 1 × 10^−7^ cm/s. The permeability coefficient of cement test blocks needed to be tested to evaluate whether it meets the impermeability standards of the tailing ponds. We designed the experiments for the permeability coefficient of the cement test blocks after mixing the cement slurry with the tailing sand consolidation. The experiments were conducted using the TC-50 core-flooding apparatus for the cement [31]. This system included a core chamber where the core samples were loaded to apply upstream pressure (overburden pressure), a hand-operated pump to control the confining pressure, and two constant speed and constant pressure pumps to inject the Deionized water solutions stored in three different plunger containers into the core samples. The dynamic variations in the upstream pressure and permeability were monitored at 60 s via a pressure transducer and balance with an accuracy of 0.001 g (Figure 3).

Firstly, cement core samples with a diameter of 25 mm were drilled using a core drilling machine, and their surfaces were polished with a metallographic specimen grinding machine, cleaned, and dried in an electric blast dryer, then the length of the core was measured. After that, the core was placed into a core holder; the confining pressure was set to 5 MPa. The constant flow mode was set, the working fluid inflow flow rate *Q* was 0.5 mL/min, and when there was continuous liquid seepage from the outlet, we could detect the real-time permeability of the cement core sample until a certain volume of liquid was injected. The permeability of the cement core sample was calculated according to Darcy’s law (Equation (1)).

(1) $$k=\frac{Q\mu L}{A(P_{2}-P_{1})}$$

where *Q*: the flow rate of the fluid through the core per unit time, cm^3^/s;

*μ*: viscosity of the liquid at the experimental temperature, mPa·s;

*L*: length of the core, cm; *P*_2_: inlet pressure, MPa; *P*_1_: outlet pressure, MPa;

*A*: the cross-sectional area of the fluid through the core, cm^2^.

The permeability coefficient of the cement core is calculated using the following formula (Equation (2)):

(2) $$K=k\times \frac{r}{\mu }$$

where *K*: permeability coefficient of the cement core, cm/s;

*k*: permeability of the cement core, mD;

*r*: volumetric weight of water, kN/m^3^;

*μ*: dynamic coefficient of the viscosity of water, Pa·s.

#### 2.2.5. Compressive Strength Test

The compressive strength test was used to evaluate whether the consolidation body mixed with the tailing and the high-pressure rotary jet cement slurry incorporated into the tailing’s stratum met the strength requirements. According to the “Standard of Geotechnical Test Method” (GB/T 50123-2019), the experiment used a computer-controlled compressive and flexural strength testing machine, and the unconfined compressive strength of the cement slurry mixed with tailing was detected.

#### 2.2.6. Evaluation of Heavy Metal Solidification Effect of Slurry

To investigate the positive effect of admixtures on heavy metal elements present in the tailing sand, cement tailing sand solids were created, and soaked in deionized water for 28 d, the soaking solution was obtained, and nitric acid was added to bring the solution to a neutral salt solution, 10 mL of filtrate was obtained using a syringe with a 0.45 μm filter membrane, and the elemental concentrations of Cr, Cu, Zn, As, Cd, and Pb in the solution were measured by ICP-MS.

#### 2.2.7. Micro-Morphological Analysis

After following these experiments conducted on the tailing sand using different cement slurries, only the result following surface consolidation could be observed, and it was difficult to observe the formation of internal fissures in the cement specimens after consolidation and whether the tailing sand was completely cured. Environmental scanning electron microscopy (SEM) and energy spectrometer elemental analysis (EDS) experiments were conducted to observe and analyze the specimens following the cement tailing sand consolidation. Different scales (10 μm, 20 μm, 40 μm, 50 μm, 100 μm) were selected for the same sample to observe the surface fissures and take 1~2 images, and the maximum fissures in the images were measured using ImageJ. Additionally, point scanning near the fissure for EDS element analysis.

## 3. Results and Discussions

### 3.1. Environmental Investigation of the Tailing Pond and the Analysis of the XRD Results of Tailing Sand Samples

According to the results of the soil samples obtained by the Beijing Mining and Metallurgy Technology Group Co., Ltd., the soil obtained from around the tailing pond was generally weakly acidic with an average pH value of 5.35. Based on the national standard of the People’s Republic of China, “Agricultural Land Risk Management and Control Standard” (GB 15618-2018), copper, chromium, zinc, lead, cadmium, arsenic, and mercury levels in the soil collected from around the tailing pond exceeded the risk screening value. It was determined that most of the heavy metal presenting a certain type of activity in the tailing entered the surrounding soil through surface runoff or groundwater flow. Since the tailing pond was decommissioned in 2015, the tailing sand on the surface was directly exposed and was not covered, and the waste slag present in the tailing pond was washed away by rainwater, so that the heavy metal within the waste slag entered the surrounding soil, surface water, and groundwater, resulting in the excess of heavy metal in the soil located around the tailing pond. Therefore, an effective means of creating the impermeability of the base of the reservoir needs to be conducted to ensure that the tailing reservoir not be leaked.

XRD analysis was performed on three randomly taken tailing sand samples, and the relative mineral content data obtained are shown in Table 4. The XRD test was conducted by the State Key Laboratory of Geological Process and Mineral Resources, China. The quantitative analyses of the samples were performed by X-ray Run software. The main mineral compositions evident are quartz (SiO_2_), siderite (FeCO_3_), and calcite (CaCO_3_), which account for more than 70% of the total composition. The content of clay minerals is low, and some sampling points also contain ankerite (Ca(Mg, Fe)(CO_3_)_2_). The presence of these minerals can also be cross-corroborated in the EDS point-scan experiments.

### 3.2. Laboratory Research Conducted on Three Kinds of High-Pressure Rotary Jet Slurry Systems

#### 3.2.1. A “Simple” P.O 42.5 Cement System

The high-pressure rotary jet slurry formulation was designed in a laboratory, based on P.O 42.5 cement. The experimental materials used were water, P.O 42.5 cement, and lead–zinc tailing. Setting time, fluidity, compressive strength, and permeability coefficient tests were conducted for the formula with different water–cement ratios (0.7, 0.8, 0.9, and 1.0). The results are presented in Table 5.

#### 3.2.2. A “Complex” P.O 42.5 Cement Slurry System Containing a Water-Reducing Agent and Solidification/Stabilization Agents

Based on the P.O 42.5 cement, orthogonal experiments were conducted with the addition of different quick-setting and water-reducing agents, as well as experiments on the reduction of the permeability coefficient. Formulas were selected through orthogonal experiments to obtain a set of cement slurry systems suitable for the high-pressure jet grouting of the Shenxiandong tailing pond. The formulas and basic performance characteristics of the cement slurry are presented in Table 3 and Table 4. The addition of the additives is calculated relative to the quality of the cement.

The slurry system preferred in Table 3 was mixed thoroughly with the tailing sand of Shenxiandong and maintained in a water bath at 27 °C under normal pressure. The results are presented in Table 5.

According to the results (groups b–e), when the water–cement ratio is reduced to 0.7 or less, the permeability coefficient of the consolidation body is less than 1 × 10^−8^ cm/s, and the compressive strength also meets the requirements for the compressive strength of the tailing’ consolidation mass. According to the experimental results for the compressive strength and permeability coefficient, the compressive strength of the cement test block increases with the increase in curing days, and the permeability coefficient decreases with the increase in curing days. This result mainly occurs since the hydration of cement occurs in stages. In the early curing stage, this is mainly caused by the reactions of tricalcium silicate and tricalcium aluminate in the cement clinker. Although the two substances react rapidly, the formed substances are not dense and have a low-strength property. During the middle and late stages of the hydration reaction, the hydration products of dicalcium silicate have a good bonding strength capability, and the generated clusters of cementitious materials effectively fill the pores, which significantly reduces the permeability coefficient of the samples [32].

**Figure 4:**
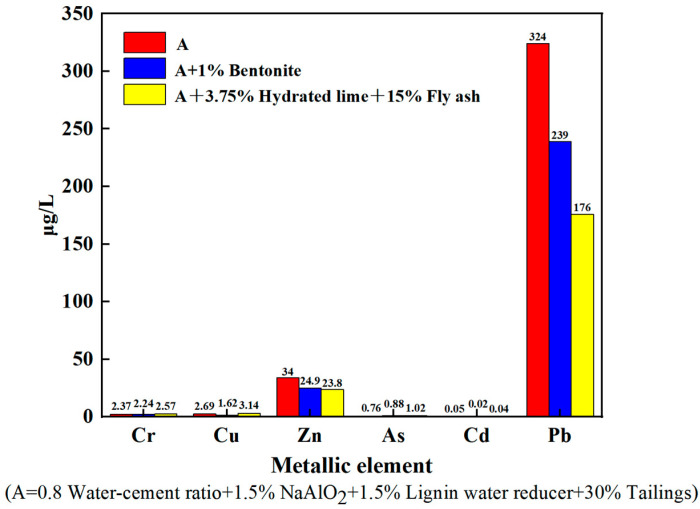
ICP-MS experimental results for group a formula.

ICP-MS experiments were performed in the group a system, and the experimental results are shown in Figure 4.

The ICP-MS experimental results show that bentonite has a passivating effect on Cu, Zn, Pb, Cd, and Cr in the tailing sand that occurs because the 2:1 crystal layer structure of bentonite gives it a large specific surface area, pore size, and pore volume, and its surface has Gibbs free energy, surface activity, and a negative surface charge, which causes bentonite to have a strong adsorption effect on heavy metal ions [33,34]. The concentration of Cu decreases by 39.8%, that of Zn decreases by 26.8%, that of Pb decreases by 26.2%, that of Cd decreases by 60%, and that of Cr decreases by 5.5%, however, it may promote the release of As. The combination of fly ash and hydrated lime has a more obvious passivation effect on Zn and Pb, and the decrease in Pb and Zn concentrations is 45.7% and 30%, respectively. Hydrated lime can improve the pH value in soil and form hydroxide precipitates [35]. Fly ash consists of numerous powdery particles, and these irregular particles share a porous surface [36,37,38,39] and therefore have an adsorption effect on heavy metal.

#### 3.2.3. Microstructure Analysis of Cement-Tailing Sand Consolidation Body

**Figure 5:**
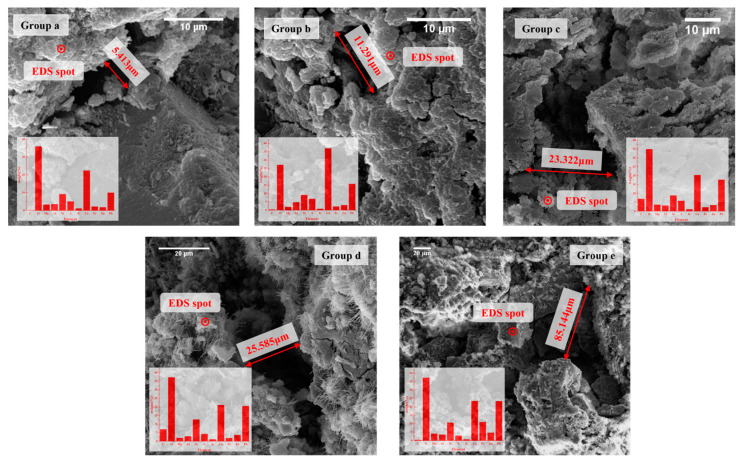
SEM images of groups a–e cement tailing consolidation test block samples.

**Figure 6:**
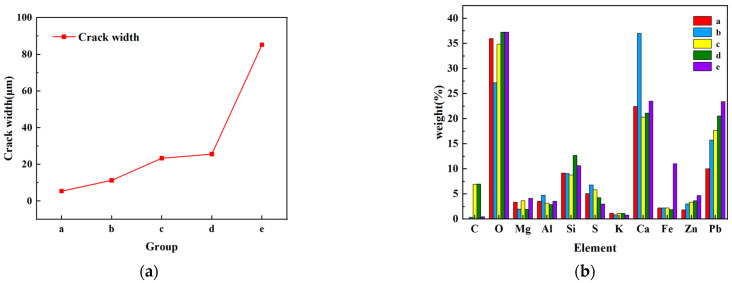
Experimental results of microstructures of groups a–e. (**a**) Crack width test results. (**b**) Point scanning electron microscope elemental composition map.

The SEM-EDS experiment was performed on the cement tailing consolidation body with the groups a–e formula, and the results are presented in Figure 5. The micro-surface crack width and EDS element quantitative analysis results are presented in Figure 6.

According to Figure 5, the maximum crack width present on the microscopic surface of the experimental cement test block of group a is 5.413 μm. The crack width of the experimental cement block of group e is more developed, which is 85.144 μm. Due to the small difference in the size of the sample cement test blocks, which were randomly selected, it was intuitively observed that there were more cracks in group e during the scanning process, while it was difficult to find cracks in the cement test blocks in the group an of formulations.

**Figure 7:**
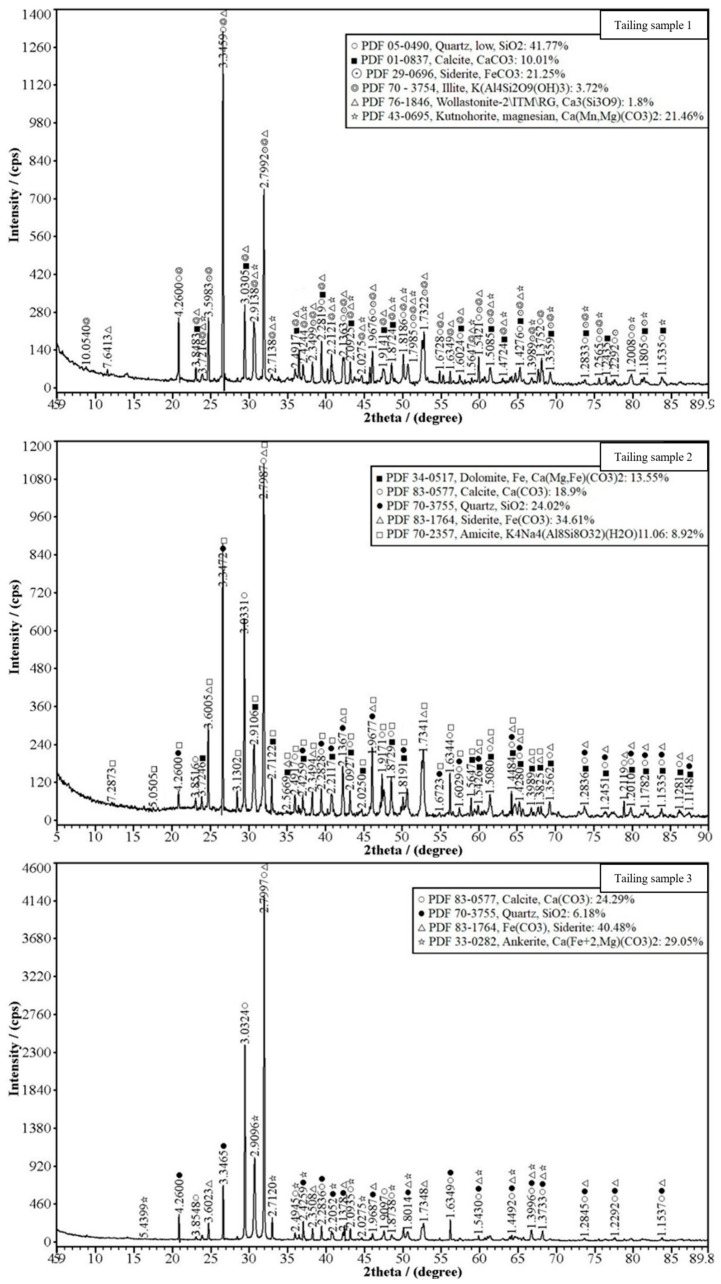
XRD spectrographs of the three tailing samples.

According to the XRD spectrographs of tailing (Figure 7) and analysis results of the element percentage in the point scanning of the five groups of cement test blocks (Figure 6b), C, O, Al, Si, Ca, K, and S elements may form part of calcium silicate, wollastonite (Ca_3_Si_3_O_9_), amicite, admixture NaAlO_2,_ and lignin water-reducing agents. The Mg element may be derived from dolomite [CaMg(CO_3_)_2_] in cement and tailing; the Fe element may be derived from siderite (FeCO_3_) and ankerite [Ca(Mg, Fe)(CO_3_)_2_] in the tailing. Comparing the histograms of groups a–e, it can also be observed that the group a formulation system with added admixtures has a passivating effect on Pb and Zn. According to the analysis of Pb and Zn, the two main metal elements present in the tailing, in the simple P.O 42.5 cement formula system, the increase in the water–cement ratio also causes the content of heavy metal in the consolidation body to increase. It was speculated that the better development of cracks may lead to the leakage of heavy metal on the surface.

#### 3.2.4. “Simple” Specially Developed Cementitious Material Slurry System

**Figure 8:**
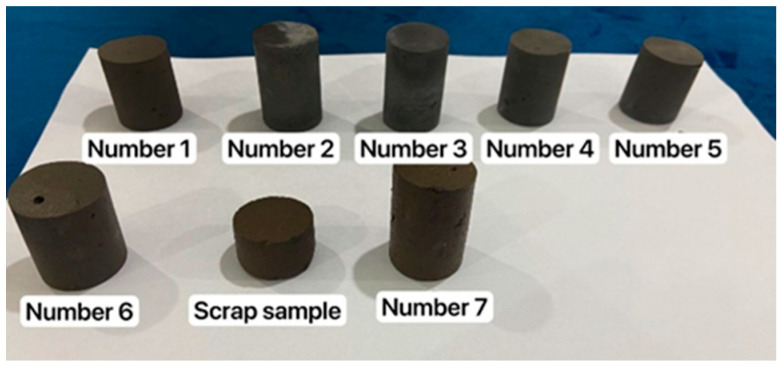
Consolidation of core samples with different formulations.

**Figure 9:**
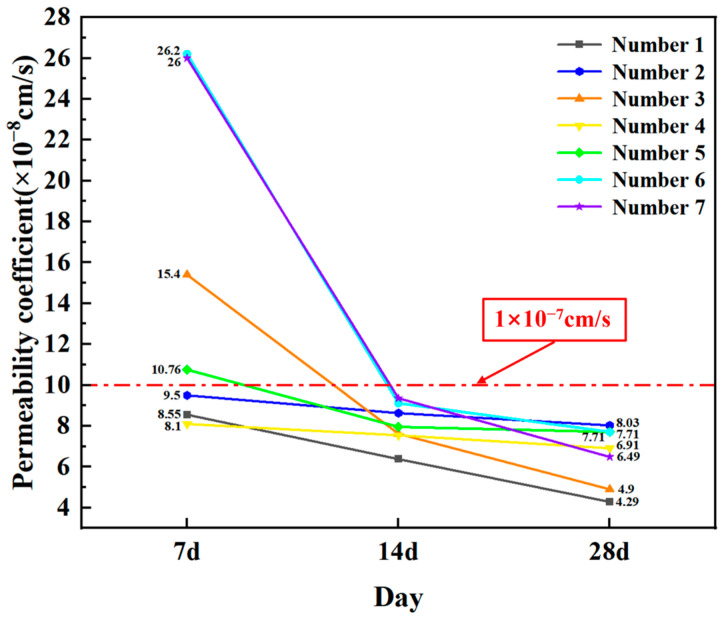
Experimental results of permeability coefficients for different formulations.

As the tailing was introduced during high-pressure rotary injecting construction, lead–zinc tailing was added to the laboratory simulation formula design. The consolidation core samples are presented in Figure 8. The experimental data of the compressive strength and permeability coefficient are exhibited in Table 6 and Figure 9.

Based on changing the water–solid ratio (the quality ratio of the water and curing agent) and tailing content, multiple groups of compressive strength and permeability coefficient experiments were conducted. According to the experimental results (Figure 9), with the increase in the curing time, the compressive strength of the consolidation body also increases, and the permeability coefficient significantly decreases, all of which are below the red-line standard at 14 d. The addition of different tailing contents was discussed: when the water–solid ratio was below 0.9 and the tailing replacement level was below 40%, the permeability coefficient reached the required value at 7 days. If the tailing content increased, the amount of curing agent needed to be increased, and the curing effect of the curing agent became increasingly stable with the increase in time, and the permeability coefficient decreased more evidently. The addition of the curing agent should be adjusted accordingly during construction. According to the requirements of the anti-seepage standard of tailing reservoirs, it can be observed that the curing agent can meet the construction requirements of the deep high-pressure rotary jet grouting technology in the Shenxiandong tailing pond and produces a good pollution barrier effect.

## 4. Field Trial of the High-Pressure Rotary Jet Method

### 4.1. High-Pressure Rotary Jet Technology

**Figure 10:**
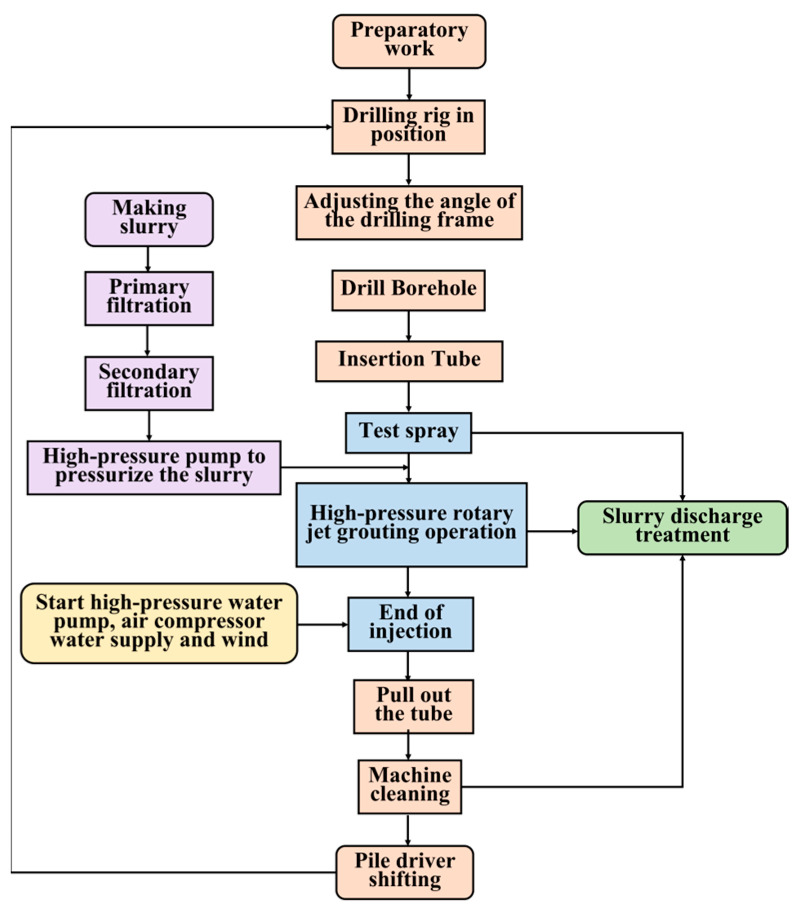
The double-pipe method process flow chart.

**Figure 11:**
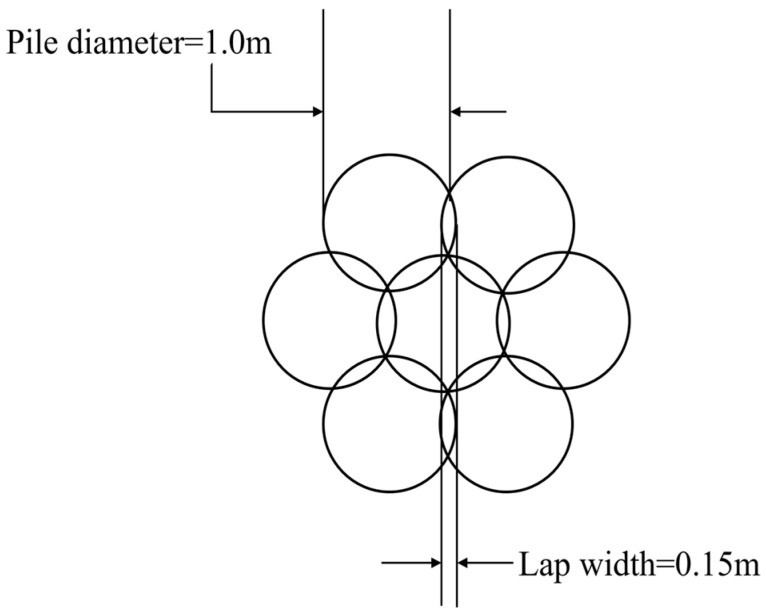
High-pressure rotary pile-placement diagram.

In 2022, the double-pipe method was adopted for use in the Shenxiandong tailing pond for seepage treatment. Its process flow chart is presented in Figure 10. The diameter of the high-pressure rotary spray pile for this project was set as 1000 mm. According to the “Technical Specification for High-Pressure Jet Grouting Construction” (HG/T 20691-2017), the lap width of the high-pressure rotary pile for this project was set as 150 mm, i.e., the pile distance was 850 mm. The layout diagram for the high-pressure jet pile is presented in Figure 11.

### 4.2. Field Trial Conducted in the Shenxiandong Tailing Pond

**Figure 12:**
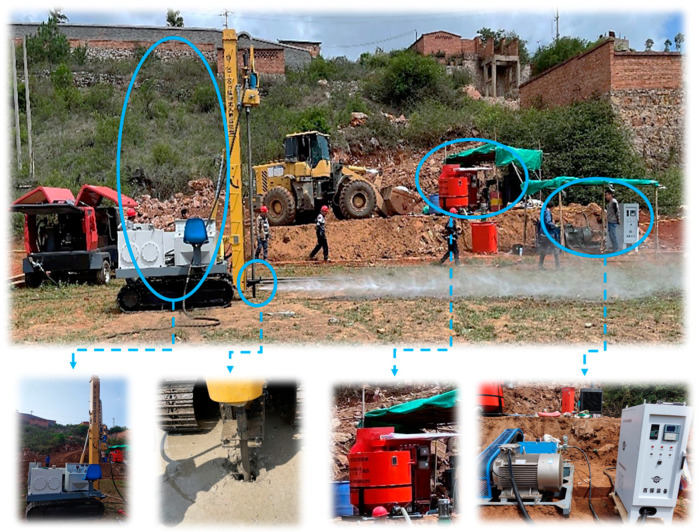
Images of high-pressure rotary jet grouting.

Using the XL-50C crawler-type high-pressure rotary jet grouting rig (Figure 12), the field trial of a high-pressure rotary spray was conducted in the Shenxiandong lead–zinc tailing pond in Jianshui County, Honghe Hani and Yi Autonomous Prefecture, Yunnan Province. The effect of high-pressure jet grouting performed on-site is presented in Figure 12. The grouting pump used at the construction site had a pumping capacity of 35 L/min, a maximum pump pressure of 30 MPa, and it was equipped with a primary and secondary mixer. Following the completion of the test pile, the water–solid ratios of 0.8, 0.9, and 1.0 were optimized. Considering the influence of the water–solid ratio on the permeability coefficient of the consolidation body in the laboratory experiment, a water–solid ratio of 0.8 and pile diameter of 1.0 m were finally determined. The pile depth was 10 m, the jet thickness was 2 m, and the dosage of cementing materials per meter was 0.42 t. The parameters of the field construction are presented in Table 7.

The high-pressure rotary jet grout density was 1.4~1.52 g/cm^3^, and the viscosity of the Markov funnel used was 34~40 s. The compressive strength of the solidification body after 28 d was 7.45 MPa, and the permeability coefficient at 28 d was 6.271 × 10^−8^ cm/s. To summarize, 2142 high-pressure rotary jet piles were constructed, covering an area of 1000 m^2^, presenting a drilling depth of 21,000 linear meters, and producing a spray distance of 4284 linear meters.

**Figure 13:**
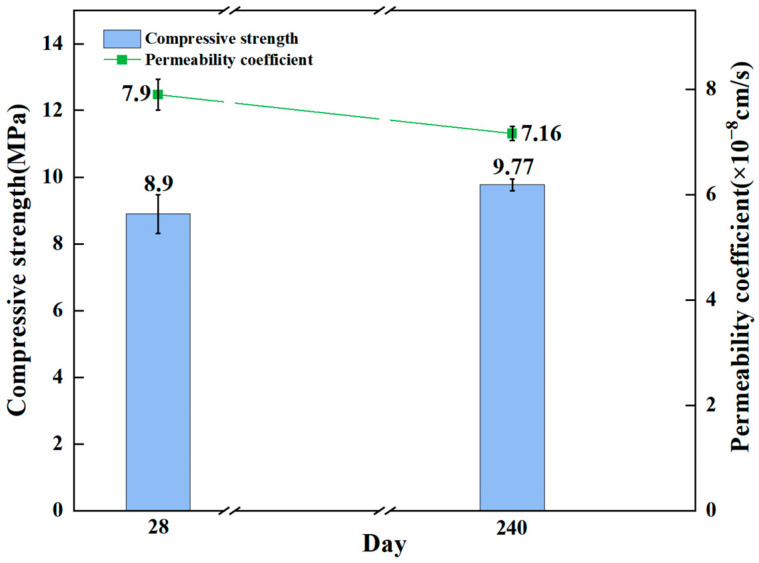
The compressive strength and permeability coefficient of field samples.

A follow-up evaluation of the demonstration construction effect of deep high-pressure rotary jet grouting technology was conducted. The samples were made by mixing a 0.8 water-to-solid ratio slurry prepared at the Shenxiandong with tailing sand equivalent to 80% of the slurry mass, collected from the field, and tested for compressive strength and permeability coefficient. The results are shown in Figure 13.

Testing of consolidation samples after 240 days’ maintenance proved that after a standard 28 days period, the compressive strength of the consolidation body was increased by 9% and the permeability coefficient was decreased by 9.4%, which is similar to recent work of E. A. Ermolovich [40]. The compressive strength, permeability coefficient, and curing time of the consolidation body are in the theoretical design requirements. This further indicated that the consolidation body formed by the high-pressure rotary jet grouting technology had a relatively stable compressive strength and a good barrier effect on heavy metal contamination control.

Groundwater samples were collected from three different locations and sent to third-party testing institutions to test the pH levels and metal element concentrations, focusing on the index concentrations of zinc, lead, cadmium, mercury, and manganese, whose groundwater pH values were 7.3–7.6, with an average value of 7.5. The heavy metal concentration indicators of key concern are presented in Table 8. Compared to the excess number of heavy metals present in the original groundwater, all groundwater indexes were less than the requirements of the corresponding limit value of the “Groundwater Quality Standard” (GB/T 14848-2017).

A surface water sample was collected and sent to a third-party testing institution to test its pH level and the concentration of metal elements present in it, focusing on the index concentrations of zinc, lead, cadmium, mercury, and manganese. The surface water pH level was 7.3. The heavy metal concentration indicators of key concern are presented in Table 9. Compared to the excess number of heavy metals in the original groundwater, all the indexes in the surface water were less than the corresponding limit values of the “Surface Water Environmental Quality Standard” (GB 3838-2002).

High-pressure rotary jet grouting technology disturbs the soil structure drastically and is suitable for shallow contamination of clayey soil with very poor permeability [41,42], so it is very informative to use this technology to solve the problem of deep reservoir bottom impermeability of Shenxiandong heavy metal tailing pond. For the uncertainties in the construction of rotary piles, corresponding countermeasures were taken, such as uneven strength of consolidation (appropriate adjustment of nozzle rotation speed, lifting speed, injection pressure, and slurry volume), drilling bubbling slurry (adding the appropriate amount of accelerator, increasing injection pressure, appropriate reduction of nozzle diameter, accelerating lifting and rotation speed), the emergence of concave cavities at the top of consolidation (drilling 0.5–1.0 m at the top of consolidation and reinjection of slurry in situ), etc. However, due to the non-homogeneity of the distribution of heavy metal in the actual tailing sand, there is uncertainty in the effect of heavy metal stabilization and consolidation, and for such problems, continuous follow-up observation of heavy metal passivation in the tailing sand should be carried out subsequently. According to the results of the previous monitoring effect assessment, the treatment and repair of the tailing pond are effective, and the heavy metal targets can basically meet the repair requirements.

## 5. Conclusions

(1) Either “simple” P.O 42.5 cement or “simple” specially developed cementitious material slurry; the permeability coefficient of the solidification body after 28 days of curing was less than 1 × 10^−7^ cm/s. For the complex P.O 42.5 cement slurry system, after 28 days of curing, the solidification body’s permeability coefficient was 3.56 × 10^−8^ cm/s and the compressive strength was 11.45 MPa.

(2) The preferred high-pressure rotary jet slurry system in the laboratory for deep tailing in the Shenxiandong tailing pond is 0.8 water–cement ratio + 1.5% NaAlO_2_ + 1.5% lignin water reducer + 1% bentonite + 3.75% hydrated lime + 15% fly ash + 33.3% tailing. This formulation system containing bentonite, slaked lime, and fly ash can passivate the heavy metal elements present in the tailing sand. This was because the 2:1 crystal layer structure of bentonite creates a surface with Gibbs free energy, surface activity, and negative surface charge, which can adsorb heavy metal ions; hydrated lime can improve the pH level in soil and form hydroxide precipitates, and the irregular particles composing fly ash have a common, porous surface, which also has an adsorption effect on heavy metal.

(3) Field trial of an XL-50C high-pressure rotary jet grouting rig was conducted in the deep seepage treatment of the Shenxiandong lead–zinc tailing pond. The performance of high-pressure rotary jet grout prepared on-site was tested; after 28 days of curing, the compressive strength was 7.45 MPa and the permeability coefficient was 6.271 × 10^−8^ cm/s, which meets the deep seepage treatment requirements of the tailing pond, i.e., the compressive strength was greater than 1 MPa and the permeability coefficient was less than 1 × 10^−7^ cm/s.

(4) Both experimental tests and field test results show that high-pressure rotary jet technology can be effectively applied to a tailing pond base impermeable. The solidification body is formed by a good pollution barrier effect, which can prevent heavy metals from spreading to adjacent soil and groundwater through karst cavities. This study provides a significant reference to the solidification and stabilization of deep contaminated sites. It has great potential that combines the high-pressure rotary jet technology with other technologies such as horizontal directional drilling in the future.

## Figures and Tables

**Table 1:** Three types of jet-grouting methods and application fields.

Types	Injected Fluids	Application Fields
Single-pipe method	Grout	Geotechnical applications (foundation reinforcement [7], impervious walls [8], bottom plugs [9], tunnels [10,11]), seepage prevention and water stoppage [12,13,14], tailing rehabilitation [15], etc.
Double-pipe method	Air and grout	
Triple-pipe method	Water or accelerator, air, and grout	

**Table 2:** High-pressure rotary jet grouting cement slurry formula for the Shenxiandong tailing pond.

Water–Cement Ratio	NaAlO_2_	Lignin Water-Reducing Agent	Bentonite	Hydrated Lime	Fly Ash
0.8	1.50%	1.50%	1%	3.75%	15%

**Table 3:** Basic properties of high-pressure rotary grout for the Shenxiandong tailing pond.

Initial Setting Time (min)	Final Setting Time (min)	Fluidity (mm)	Density (g/cm^3^)
69	323	205	1.59

**Table 4:** XRD analysis results of tailing samples 1, 2, and 3 were obtained from the Shenxiandong lead–zinc mine.

Number	Tailing Sample 1	Tailing Sample 2	Tailing Sample 3
Mineral Species			
Quartz (%)	41.77	24.02	6.18
Siderite (%)	21.25	34.61	40.48
Calcite (%)	10.01	18.90	24.29
Dolomite (%)	0	13.55	0
Kutnohorrrite, magnesian (%)	21.46	0	0
Illite (%)	3.72	0	0
Amicite (%)	0	8.92	0
Ankerite (%)	0	0	29.05
Wollastonite (%)	1.8	0	0

**Table 5:** Initial settings time, final settings time, fluidity, compressive strength, and permeability coefficient results of different formulations.

Group	Formulations	Initial/Final Settings (min)	Fluidity (mm)	Compressive Strength (MPa)	Permeability Coefficient (10^−8^ cm/s)
				7 d/28 d	7 d/28 d
a	0.8 water–cement ratio+ 1.5%NaAlO_2_ + 1.5%lignin water reducer +1% bentonite + 3.75% hydrated lime + 15%fly ash + 30%tailing	77/343	186	8.96/11.45	5.55/3.56
b	0.7 water–cement ratio + 30%tailing	550/617	193	10.09/12.09	9.76/8.7
c	0.8 water–cement ratio + 30% tailing	539/668	247	6.99/8.99	11.23/9.0
d	0.9 water–cement ratio + 30 tailing	668/746	293	4.89/6.89	14.3/10.3
e	1.0 water–cement ratio + 30% tailing	684/763	290	4.29/6.29	15.8/12.5

**Table 6:** Compressive strength test results for different formulations.

Number	Water: Curing Agent	Tailing Replacement Level * (%)	Compressive Strength 28 d (MPa)
1	0.6	34.8	5.67
2	0.75	40	4.72
3	0.75	60	3.13
4	0.9	44.4	3.15
5	1	47.1	2.49
6	1	60	2.96
7	1.5	80	2.46

* Tailing replacement level: tailing quality/total solids quality, where the total solid quality = tailing + curing agent.

**Table 7:** Construction parameters of the high-pressure rotary jet method.

Pump Volume (L/min)		Pump Pressure (MPa)	Air Pressure (MPa)	Lifting Speed (m/min)	Rotating Speed (r/min)
Drilling process: 4	Rotary jet process: 35	30	0.7	0.15	20

**Table 8:** Heavy metal effect indicator data sheet.

Heavy Metal Indicators	Original Mean Values (μg/L)	Appraisal Criteria (μg/L)	Detection Limits (μg/L)	Concentrations (μg/L)		
				Minimum Values	Maximum Values	Average Values
Zn	13	2000	0.67	13.4	268	102
Pb	0.87	50	0.09	ND	ND	ND
Cd	0.2	5	0.04	ND	ND	ND
Hg	0.2	1	0.04	ND	ND	ND
Mn	526	2000	0.12	11.4	75.3	44.6

**Table 9:** Heavy metal effect indicator data sheet.

Heavy Metal Indicators	Original Mean Values (μg/L)	Appraisal Criteria (μg/L)	Detection Limits (μg/L)	Concentrations (μg/L)
Zn	2190	5000	0.67	67.9
Pb	16.3	180	0.09	ND
Cd	24.9	10	0.05	ND
Hg	0.1	2	0.04	0.38

## Data Availability

Some or all data that support the findings of this study are available from the corresponding author upon reasonable request.
